# Achieving functional remission in schizophrenia: a pilot study

**DOI:** 10.1192/j.eurpsy.2024.1513

**Published:** 2024-08-27

**Authors:** D. Bošnjak Kuharić, A. Jambrošić Sakoman, Z. Lovrić Makarić, A. Savić, A. Papić, M. Herceg

**Affiliations:** ^1^Department for psyhotic disorders- female, University Psychiatric Hospital Vrapče; ^2^Division for Epidemiology of Communicable Diseases, Croatian Institute of Public Health; ^3^Department for first episode psychosis, University Psychiatric Hospital Vrapče, Zagreb, Croatia

## Abstract

**Introduction:**

Many patients with schizophrenia are unable to achieve adequate levels of psychosocial functioning and quality of life despite of the remission of illness symptoms. According to previous reports, only one-third of patients with symptomatic remission reach functional remission. While current pharmacotherapy options seem to be relatively effective for different symptoms of schizophrenia (e.g. positive symptoms), more specific psychosocial interventions that could enable functional remission are yet to be developed.

**Objectives:**

Our objective is to investigate differences in psychopathology, quality of life, functioning, and achieving functional remission before and after specific group treatments developed in our clinic.

**Methods:**

We will conduct a prospective study including a consecutive cohort of female patients older than 18 years of age, which fulfilled the criteria for schizophrenia and schizoaffective disorder according to the International Classification of Disorders, 10th revision. Exclusion criteria are intellectual disabilities, mental disorders due to known physiological or neurological conditions, lactation or pregnancy, treatment with medications that can provoke psychosis, alcoholism, and other addictions. Patients will be recruited after finished hospital treatment or during individual outpatient controls. The Recovery Helm will be used at the beginning of the treatment, to make individual treatment plan and include patients in specific programs including day hospital treatment and/or outpatient group programs: psychoeducation, relaxation, metacognitive training, and social skills training. Besides collecting sociodemographic data, pre- and post-treatment assessment will include the Positive and Negative Syndrome Scale (PANSS), the Global Assessment of Functioning (GAF), the Quality of Life Scale (QLS), and the “Functional Remission of General Schizophrenia” (FROGS) scale.

**Results:**

We will analyze the changes in psychopathology levels, quality of life, functioning, and achieving functional remission between the two assessment points, taking into account different treatment possibilities.

**Conclusions:**

Evaluation of current available programs can help with recognition of specific needs of patients with schizophrenia and provide guidelines for further development of treatment programs that could be helpful in achieving functional remission.

**Disclosure of Interest:**

None Declared

